# Evaluation of microsatellite instability in tumor and tumor marginal samples of sporadic colorectal cancer using mononucleotide markers

**DOI:** 10.17179/excli2018-1455

**Published:** 2018-09-24

**Authors:** Jafar Nouri Nojadeh, Shahriar Hashemzadeh, Hossein Samadi Kafil, Shahin Behrouz Sharif, Amirtaher Eftekharsadat, Tohid Ghasemnejad, Mortaza Ghojazadeh, Ebrahim Sakhinia

**Affiliations:** 1 Department of Medical Genetics, Faculty of Medicine, Tabriz University of Medical Sciences, Tabriz, Iran; 2 Department of General and Thoracic Surgery, Tabriz University of Medical Sciences, Tabriz, Iran; 3 Tuberculosis and Lung Disease Research Center, Tabriz University of Medical Sciences, Tabriz, Iran; 4 Drug Applied Research Center, Tabriz University of Medical Sciences, Tabriz, Iran; 5 Department of Molecular Medicine, Pasteur Institute of Iran, Tehran, Iran; 6 Department of Pathology, Imam Reza Hospital, Tabriz University of Medical Sciences, Tabriz, Iran; 7 Liver and Gastrointestinal Disease Research Center, Tabriz University of Medical Sciences, Tabriz, Iran; 8 Connective Tissue Research Center, Tabriz University of Medical Sciences, Tabriz, Iran

**Keywords:** CRC, MSI, DNA MMR system

## Abstract

Microsatellite instability (MSI) is a unique molecular alteration that is due to a defective DNA mismatch repair (MMR) system. Approximately, 15-20 % of sporadic colorectal cancers (CRC) display MSI. Determination of MSI status in CRC has prognostic and predictive implications. Additionally, detecting MSI is used diagnostically for tumor detection and classification. The present study analyzed a panel of five mononucleotide markers, BAT-25, BAT-26, NR-21, NR-22 and NR-27, amplified in a single multiplex PCR reaction to evaluate MSI status in CRC patients. Genomic DNA from 50 CRC and paired adjacent normal tissues was used for PCR-based MSI analysis. Our finding showed microsatellite instability in 36 % of specimens. Instability with differences in allele lengths was observed in the tumoral DNA compared to the tumor-free margin DNA sample. The frequency of instability in NR-21, BAT-26 and BAT-25 markers were more than others; their frequency were 35.48 %, 29.03 %, and 22.58 %, respectively. In conclusion, the NR-21, BAT-26, and BAT-25 were the most useful markers for discriminating cancer tissue from normal, therefore these markers have demonstrated promising potential for determining MSI status in patients with sporadic colorectal cancer.

## Introduction

Colorectal cancer (CRC) is the third most common cancer in humans and the third leading cause of cancer related deaths in both genders, which contributes to a major public health problem worldwide (Jemal et al., 2011[13]; Siegel et al., 2012[21]). As well, CRC is the third and fourth generally diagnosed cancer in Iranian men and women, respectively (Mahmodlou et al., 2012[16]). The absence of clinical symptoms in patients with CRC until the post-cancer stage is one of the most common hallmarks of the disease, which leads to poor prognosis and high mortality (Behrouz Sharif et al., 2016[3]). Colorectal cancer is mainly developed through the gradual accumulation of genetic and epigenetic changes in the genome (Fearon and Vogelstein, 1990[11]).

Sporadic colorectal cancer is the most common type of CRC and includes approximately 75 % of cases in which there is no obvious evidence of the inherited disorder. However, it seems that the genetic factors are not definite and the possibility of cancerous effects exists even in the absence of specific mutations (Arvelo et al., 2015[2]). There are several molecular changes in CRC such as Chromosomal Instability (CIN), Microsatellite Instability (MSI), and CpG Island Methylator phenotype (CIMP) (Worthley and Leggett, 2010[25]). Most CRCs are developed via the CIN pathway, while 15-20 percent of CRC cases represent MSI (Vilar and Gruber, 2010[24]; Cancer Genome Atlas Network, 2012[7]).

Microsatellites are short tandem repeat (STR) stretches of DNA sequence distributed throughout the coding and non-coding regions of the genome, which are susceptible to high mutation rates due to their repeated structures (Ellegren, 2004[9]). Microsatellite instability (MSI) is a molecular phenotype rising from faulty DNA mismatch repair (MMR) system (Yamamoto and Imai, 2015[27]). DNA mismatch repair system corrects fallacious deletion, insertion, and base mismatches produced within DNA replication and recombination that have escaped the proofreading process (Jiricny, 2006[14]). MSI in tumoral DNA is defined by the presence of intermittent sized repetitive DNA sequences which do not exist in the corresponding germ-line DNA. The presence of MSI in the colon, gastric, endometrial and the majority of other sporadic cancers have been indicated (Yamamoto and Imai, 2015[27]). Determining the status of MSI in CRC has prognostic and therapeutic outcomes. Additionally, MSI clinically can be used for detection of patients with germline defects due to MMR-deficiency and is used for tumor diagnosis and classification (Setaffy and Langner, 2015[20]). 

MSI is indirectly detected by immunohistochemical staining (IHC) by analyzing the MMR protein expression, or directly with a specific microsatellite repeats amplification by PCR-based methods (Buecher et al., 2013[5]). At first attempt to detect MSI status in CRC using the PCR-based methods, which are the most common detection ways, the National Cancer Institute (NCI) suggested a five panel of microsatellite markers included three dinucleotide repeats (D5S346, D2S123, and D17S250) and two mononucleotide repeats (BAT25 and BAT26) (Rodriguez-Bigas et al., 1997[19]). After a while, it was found that mononucleotide markers are more specific and more sensitive than dinucleotide repeats since dinucleotide markers have a polymorphic nature (Suraweera et al., 2002[22]) and thus, NCI revised the Bethesda guideline criteria (Umar et al., 2004[23]).

Nowadays, the use of panels containing mononucleotide markers has increased with respect to their higher sensitivity and specificity for detecting MSI in CRCs (Buhard et al., 2004[6]; Xicola et al., 2007[26]; Agostini et al., 2010[1]; Goel et al., 2010[12]; You et al., 2010[28]; Cicek et al., 2011[8]; Nojadeh et al., 2018[18]). For this reason, our objective in the present study is to analyze a panel of five mononucleotide markers (BAT-25, BAT-26, NR-21, NR-22 and NR-27) amplified in a single pentaplex PCR reaction to evaluate their combined potential for diagnosing MSI status in CRC patients.

## Materials and Methods

### Study design and patients

This cross-sectional study was conducted as a cooperation between Tuberculosis and Lung Disease Research Center of the Tabriz University of Medical Science, Amiralmomenin and Imam Reza Hospitals, Tabriz, Iran. Study participants were Iranians who were confirmed with colorectal cancer on the basis of clinicopathological findings and all of the patients were candidates for cancer surgery. Cases that have undergone chemotherapy or radiotherapy treatments before surgery and have other malignancies, were not included in the study. The study was comprised of 22 males (44 %) and 28 females (56 %) with a median age of 59 years (range, 29-83 years). The ethical protocol of this study was confirmed by the Ethics Committee of Tabriz University of Medical Sciences and written informed consent was obtained from all patients to participate in this study. 

### Tissue specimens

Fresh tumor and tumor-free margine tissue samples were obtained from 50 sporadic colorectal cancer patients who underwent the appropriate surgical operation as a routine treatment procedure at Amiralmomenin and Imam Reza Hospitals from 2015 to 2017. After resection, the specimens were immediately snap frozen in liquid nitrogen and stored at -80 °C until further steps. The tissue samples were processed for routine histological and pathological examination by the pathologist and were divided into two different groups of 50 tumor samples and 50 margin samples. The patients' clinicopathological and demographic data were collected retrospectively.

### DNA extraction

**Figure 1:**
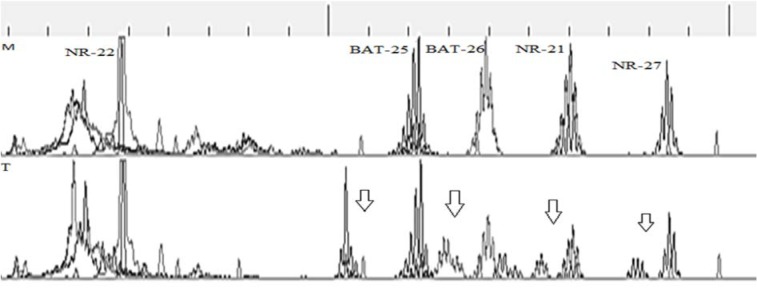
A representative graph of MSI-H profiles obtained with the pentaplex panel (NR-21, BAT-26, BAT-25, NR-22, and NR-27) in tumor (T) and matched tumor marginal (M) tissues. It represents instability for the 4 microsatellite markers (arrows), while no apparent microsatellite instability was detected in tumor marginal tissue samples in this study. BAT-26, NR-27, and NR-22 were labeled with FAM, NR-21 and BAT-26 were labeled with HEX. Abbreviations: M= tumor-free margine tissue; T= tumor tissue.

Genomic DNA was extracted separately from tumor and normal tissue samples using a standard proteinase-K and Phenol-chloroform method. The concentration of the extracted DNA and quality of the amplifiable DNA was measured by NonoDrop spectrophotometer and agarose gel electrophoresis, respectively (Figure 1(Fig. 1)). Accordingly, extracted DNA samples which had the final concentration of >100 ng/µl and optical density (OD260/280) ratio in the range of 1.7-1.9 were selected for further analysis.

### PCR reaction and MSI detection

MSI was determined by comparing the different lengths of specific microsatellite markers in tumor cells with their matched adjacent non-cancerous cells using five mononucleotide microsatellite repeats including BAT25, BAT26, NR27, NR21, and NR22. The 5' anti-sense primers were end-labeled with a fluorescent dye (6-FAM or HEX). The primers used for amplification of microsatellite markers were those used in different previous studies (17, 25-26). Primer sequences are presented in Table 1(Tab. 1).

Multiplex PCR was carried out in total volume of 25 µl containing 12.5 µl Master Mix (RED), 2.4 µl double distilled water, 1.6 µl DNA and 6 µl primers (0.96 µl of each BAT25 and BAT26; 1.44 µl of each NR22 and NR27; 1.2 µl of NR21). The PCR reactions consisted of an initial 10 minutes for denaturation step at 94 °C, followed by 30 continuous cycles at 94 °C for 15 seconds, 52 °C for 30 seconds and 72 °C for 30 seconds, with the last extension at 72 °C for 5 minutes. The amplified products were then electrophoresed on 2 % agarose gel to control the precise size and specificity. Subsequently, the fluorescent PCR products were analyzed by capillary electrophoresis using an ABI 3730XL sequencer (Applied Biosystems) and Genemapper analysis software. Tumors with instability at two or more of five markers compared with adjacent normal tissue were considered MSI-high (MSI-H), whereas those with instability at only one marker were considered MSI-low (MSI-L). Moreover, tumors with no apparent instability at any of these markers were considered Microsatellite Stable (MSS).

### Statistical analysis

The Chi-square test was used to calculate the non-parametric data distribution. Statistical analysis was performed in each group using the Mann-Whitney U test. The data obtained in this study were analyzed by descriptive statistics (frequency-percentage) and binomial test. In all tests, P value<0.05 was considered statistically significant. All analysis were performed using the SPSS version 17. 

## Results

### Clinicopathological features

The patients ranged in age from 29 to 83 years, the mean height and weight of patients were about 165 cm and about 71 kg, respectively. Twenty-two of the patients were male. There was no family history of colorectal cancer in any of the patients. The tumors were located in the right colon, transverse colon, left colon, sigmoid colon, cecal and rectosigmoid regions. The mean size of tumors was 5.6 cm (range 3-19 cm). Nine cases were Stage I, 20 were Stage II, 15 were Stage III, and 6 cases were Stage IV. Only 8 cases were active smokers. The pathological features of samples are displayed in Table 2(Tab. 2). There was no statistically significant correlation between MSI status with clinicopathological features of patients.

### MSI analysis

**Figure 2:**
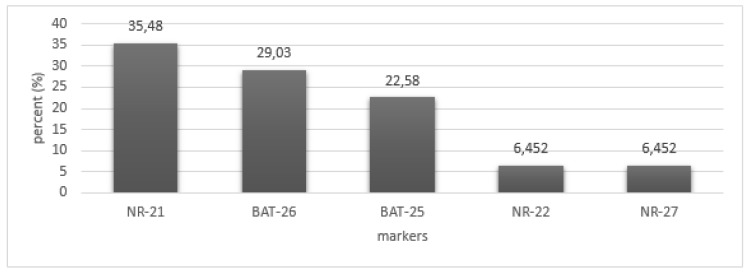
The frequency of microsatellite instability of markers

MSI was observed in 36 % of the patients. Out of 50 tumors included in the study, 8 (16 %) showed instability at only one marker (MSI-L) and 10 (20 %) were MSI-H with instability at two or more than two markers (Figure 1(Fig. 1)). Tumor free margin samples demonstrated the absence of MSI regarding investigated markers. There was no significant relationship between clinical and pathological characteristics of patients with instability in tumor sites. Our finding demonstrated that the frequency of NR-21, BAT-26, and BAT-25 markers were 35.48 %, 29.03 %, and 22.58 %, respectively. Furthermore, our results showed that the frequency of NR-22 and NR-27 markers were similar (Table 3(Tab. 3)) (Figure 2(Fig. 2)).

## Discussion

The aim of the present study was to analyze a panel of five mononucleotide microsatellite markers, BAT-25, BAT-26, NR-21, NR-22 and NR-27, amplified in a single multiplex PCR reaction to evaluate MSI status in sporadic CRC patients.

Approximately, 15-20 % of sporadic colorectal cancers and up to 90 % of Lynch syndrome (LS) patients display MSI (Goel et al., 2010[12]). In this study, the frequency of MSI in sporadic colorectal cancer was detected in 36 % of specimens which was higher than previous reports (Goel et al., 2010[12]). However, in another study performed in North-East Iran MSI was detected in 45 % of patients (Esmailnia et al., 2014[10]). 

Three different MSI phenotypes by Bethesda guideline are described. If two or more microsatellite markers are mutated, the tumor is considered MSI-high (MSI-H); if only one has mutated, the tumor is defined as MSI-low (MSI-L); and if none of the loci show instability, the tumor is considered Microsatellite Stable (Rodriguez-Bigas et al., 1997[19]). In the present study, 20 % of patients were MSI-H which is similar to the previous report (Esmailnia et al., 2014[10]).

Our investigation demonstrated that NR-21 had the highest sensitivity with 35.48 % in our patients. Furthermore, previous studies in the Iranian and Slovenian population were compatible with our result for this marker (Berginc et al., 2009[4]; Goel et al., 2010[12]). We observed the frequency of NR-22 and NR-27 to be the least and the same among the markers used; these data were compatible with Berginc et al. findings in 2009 (Berginc et al., 2009[4]). BAT-25 was the most sensitive marker in the different studies by Leite et al. (2010[15]) and Montazer Haghighi et al. (2010[17]) while in our study it was not the most instable marker.

Our study showed that the NR-21, BAT-26 and BAT-25 markers were the strongest markers for the detection of sporadic colorectal cancer, respectively, among the five markers used. Therefore, it seems that the use of a triple panel with three unstable markers can be used to determine the status of microsatellite instability in sporadic colorectal cancer patients. However, for the final confirmation, the ability of these markers to act as a promising diagnostic marker seems to be a necessity for further studies.

## Figures and Tables

**Table 1:**
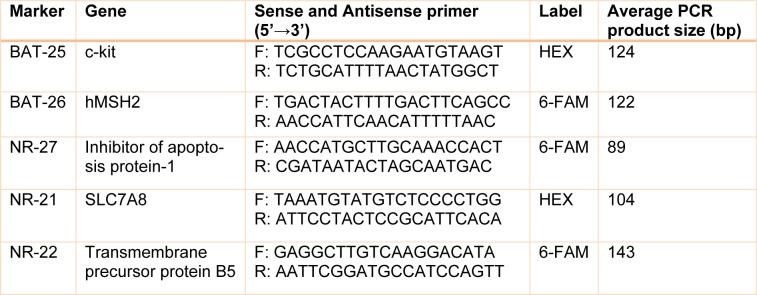
primers used for MSI assay

**Table 2:**
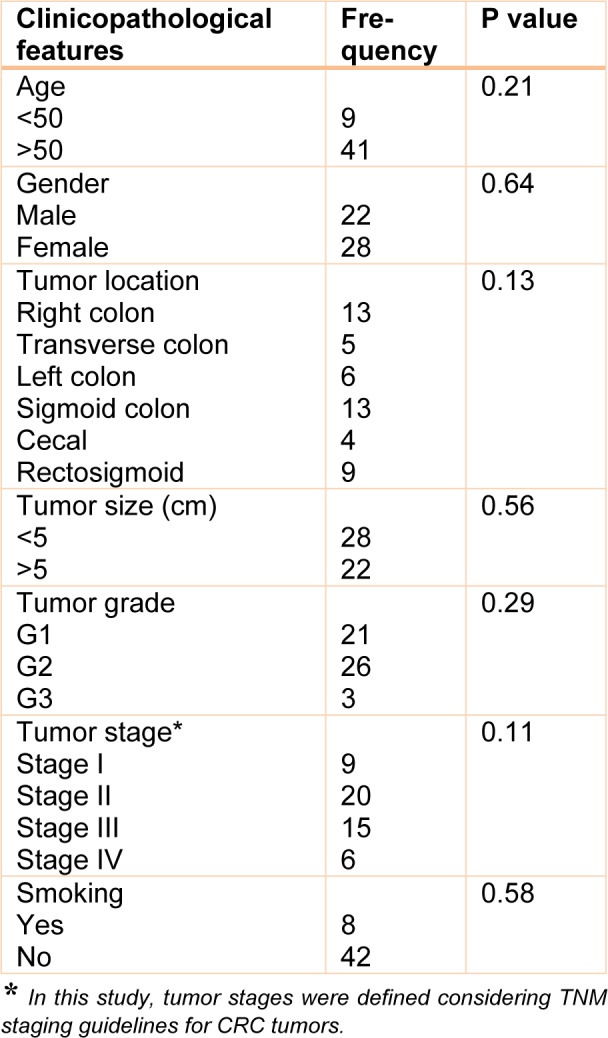
Clinicopathological findings of patients

**Table 3:**
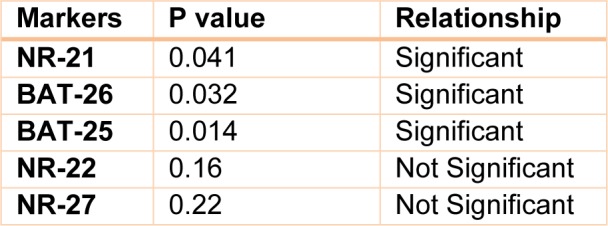
The P value of five markers compared to each other
